# Deep learning-based Intraoperative MRI reconstruction

**DOI:** 10.1186/s41747-024-00548-9

**Published:** 2025-02-25

**Authors:** Jon André Ottesen, Tryggve Storas, Svein Are Sirirud Vatnehol, Grethe Løvland, Einar Osland Vik-Mo, Till Schellhorn, Karoline Skogen, Christopher Larsson, Atle Bjørnerud, Inge Rasmus Groote-Eindbaas, Matthan W. A. Caan

**Affiliations:** 1 Computational Radiology & Artificial Intelligence (CRAI) Research Group, Division of Radiology and Nuclear Medicine, Oslo University Hospital, Oslo, Norway; 2 Department of Physics, Faculty of Mathematics and Natural Sciences, University of Oslo, Oslo, Norway; 3 Division of Radiology and Nuclear Medicine, Department of Physics and Computational Radiology, Oslo University Hospital, Oslo, Norway; 4 The Intervention Centre, Oslo University Hospital, Oslo, Norway; 5 Department of Optometry, Radiography and Lighting Design, University of South-Eastern Norway, Drammen, Norway; 6 Department of Health Sciences Gjøvik, Faculty of Medicine and Health Sciences, NTNU, Gjøvik, Norway; 7 Vilhelm Magnus Laboratory, Department of Neurosurgery, Oslo University Hospital, Oslo, Norway; 8 Institute for Clinical Medicine, Faculty of Medicine, University of Oslo, Oslo, Norway; 9 Department of Neurosurgery, Oslo University Hospital, Oslo, Norway; 10 Department of Radiology, Vestfold Hospital Trust, Tønsberg, Norway; 11 Biomedical Engineering and Physics, Amsterdam UMC, University of Amsterdam, Amsterdam, Netherlands

**Keywords:** Artifacts, Brain neoplasms, Deep learning, Magnetic resonance imaging, Neurosurgical procedures

## Abstract

**Background:**

We retrospectively evaluated the quality of deep learning (DL) reconstructions of on-scanner accelerated intraoperative MRI (iMRI) during respective brain tumor surgery.

**Methods:**

Accelerated iMRI was performed using dual surface coils positioned around the area of resection. A DL model was trained on the fastMRI neuro dataset to mimic the data from the iMRI protocol. The evaluation was performed on imaging material from 40 patients imaged from Nov 1, 2021, to June 1, 2023, who underwent iMRI during tumor resection surgery. A comparative analysis was conducted between the conventional compressed sense (CS) method and the trained DL reconstruction method. Blinded evaluation of multiple image quality metrics was performed by two neuroradiologists and one neurosurgeon using a 1-to-5 Likert scale (1, nondiagnostic; 2, poor; 3, acceptable; 4, good; and 5, excellent), and the favored reconstruction variant.

**Results:**

The DL reconstruction was strongly favored or favored over the CS reconstruction for 33/40, 39/40, and 8/40 of cases for readers 1, 2, and 3, respectively. For the evaluation metrics, the DL reconstructions had a higher score than their respective CS counterparts for 72%, 72%, and 14% of the cases for readers 1, 2, and 3, respectively. Still, the DL reconstructions exhibited shortcomings such as a striping artifact and reduced signal.

**Conclusion:**

DL shows promise in allowing for high-quality reconstructions of iMRI. The neuroradiologists noted an improvement in the perceived spatial resolution, signal-to-noise ratio, diagnostic confidence, diagnostic conspicuity, and spatial resolution compared to CS, while the neurosurgeon preferred the CS reconstructions across all metrics.

**Relevance statement:**

DL shows promise to allow for high-quality reconstructions of iMRI, however, due to the challenging setting of iMRI, further optimization is needed.

**Key Points:**

- iMRI is a surgical tool with a challenging image setting.
- DL allowed for high-quality reconstructions of iMRI.
- Additional optimization is needed due to the challenging intraoperative setting.

**Graphical Abstract:**

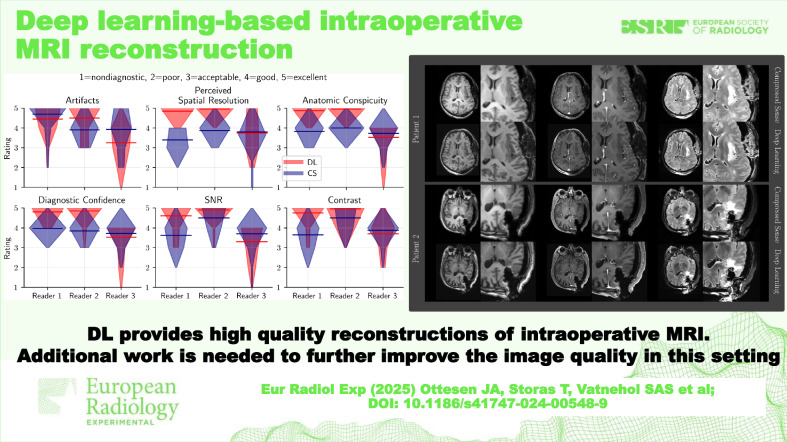

## Background

Intraoperative magnetic resonance imaging (iMRI) provides surgeons with updated images to improve the maximal safe resection of brain tumors, allowing for a greater extent of resection without an increase in adverse effects [1–3].

Fast imaging times and high image quality are essential to optimize treatment outcomes and minimize the idle time of surgical staff during the iMRI protocol. The suboptimal conditions for imaging under which iMRI is performed extend both acquisition time and reduce image quality. Manually placed surface coils prohibit fast parallel imaging through generalized autocalibrating partially parallel acquisitions or sensitivity encoding [4, 5] that otherwise accelerates brain MRI. The use of iMRI has increased the duration of surgery from 20 min to 60 min depending on the imaging protocol and surgical setup [6, 7]. Additionally, the images are more prone to noise distant from the coil elements, unlike those acquired using conventional head coils. Since its advent, one of the main strides in iMRI has been the improvement of image quality, ranging from low to high-field scanner configurations [8]. Recent reports highlight the need voiced by surgeons for improved iMR image quality [9].

To address prolonged scan times, compressed sense (CS) [10] may be adopted in iMRI. Still, substantial undersampling leads to image artifacts, poor signal-to-noise ratio (SNR), lower resolution, and potentially non-diagnostic or poor-quality images where the surgically accessible tumor is not identified. A promising alternative is the utilization of deep learning (DL) for reconstruction, aiming to accelerate MRI and enhance quality through denoising and super-resolution strategies. Key to its success is training DL networks on relevant MRI data that is representative of the foreseen imaging setting [11–14]. Initial prospective studies on knee, spine, and brain images have demonstrated that DL networks were able to generalize anatomy and pathology beyond their training scope [15–18]. Further evidence is needed to warrant deploying DL networks in the highly variable iMRI setting.

In this retrospective study, we aimed to investigate: (i) the generalizability of DL-based reconstruction on on-scanner accelerated iMRI during tumor resection surgery after being trained on conventional neuro MRI; and (ii) whether DL-based reconstruction can generate high-resolution iMR images with improved diagnostic quality compared to the currently used protocol and ease the detection of tumor residuals. We adopted the densely interconnected residual cascading network [19] and trained the network on the fastMRI neuro dataset [20, 21] with pairs of fully sampled and retrospectively downsampled images, adjusted to match the clinical protocols’ downsampling scheme. We hypothesized that DL-based reconstruction could successfully generalize to an intraoperative protocol and outperform the current standard of the CS method^1^.

## Methods

### Participants

This retrospective study was approved by the Regional Medical Ethics Committee for Oslo University Hospital (REK 367336), and informed consent was acquired prior to surgery according to a broad research approval and biobank (REK 2016/17091). Only patients over 18 years of age were included.

This study cohort included 40 patients (mean = 53 years, standard deviation = 14 years, 17 women), with the following histopathology based on the fifth edition of WHO classification of tumors of the central nervous system [22]: glioblastoma (*n* = 18); astrocytoma (*n* = 9; 2 grade 2, 4 grade 3, and 3 grade 4); oligodendroglioma (*n* = 9; 7 grade 2 and 2 grade 3); dysembryoplastic neuroepithelial tumor (*n* = 1), brain metastases from a malignant melanoma (*n* = 1); lymphoma (*n* = 1), and tumor with bleeding without confirmed diagnosis due to biopsy quality (*n* = 1).

### Acquisition protocol

Imaging was performed on a Philips Ingenia 3-T MRI using two receiver surface coil elements (Philips Healthcare, Best, The Netherlands). Patients were head clamped and fixed to the operating table. Positioning had to take into consideration the surgeon’s access to the tumor, adequate access for airway management, and the comfort of the patient (for those who were operated upon with awake monitoring), in addition to the position within the gantry. Most patients were positioned well outside the imaging isocenter. During surgery, the surgical tabletop was translocated to the MRI situated in the room next door.

The imaging protocol included sagittal three-dimensional T1-weighted before and after intravenous contrast injection (0.1, dose mmol/kg, Bayver AB, Sweden) and three-dimensional T2-weighted fluid-attenuated inversion-recovery (FLAIR) image sets. Additionally, two-dimensional axial T2-weighted turbo spin-echo and diffusion-weighted MRI were acquired, but not included in the study due to difficulties in the export of the raw k-space data. The pre- and post-contrast T1-weighted image sets were acquired with a 135–153 × 199 × 384 acquisition matrix with a resolution of 1.25 × 1.24 mm^2^ and a slice thickness of 1.74 mm (scan time 3:56 min:s). The T2-weighted FLAIR images were acquired with a 119–133 × 165 × 336 acquisition matrix with a resolution of 1.49 × 1.50 mm and a slice thickness of 1.50 mm (scan time 5:26 min:s), and two excitations. Of note, due to scanner settings, certain patient orientations resulted in an extended field-of-view (FOV) oversampling factor of approximately $\sim$2.8 along the posterior–anterior direction, with an equal factor of undersampling. In-plane oversampling occurred for 30 of the 40 patients, altering their acquisition matrix to 135–153 × 569 and 119–133 × 165 for the T1-weighted and T2-weighted FLAIR images, respectively. The FOV was extended to avoid infolding artifacts from objects outside the FOV.

K-space was sampled with a pseudorandom pattern with acceleration factors of 1.5 and 3.7 for the T1-weighted and T2-weighted FLAIR images respectively. For the images with an oversampling factor, the acceleration factors were increased to 4 and 10 for the T1-weighted and T2-weighted FLAIR images, respectively.

The on-scanner pre- and post-contrast T1-weighted reconstructions had a zero-filled interpolated reconstruction resolution of 0.78 × 0.78 mm^2^ with a slice thickness of 0.87 mm; the T2-weighted FLAIR scanner reconstructions had a resolution of 0.74 × 0.74 mm^2^ with a slice thickness of 0.75 mm. The same scans used for the CS reconstruction were also used for the DL reconstruction.

### Data processing and training

The densely interconnected residual cascading network [19] was trained to reconstruct accelerated data and to achieve super-resolution from an in-plane resolution of 1.2–1.6 mm to 0.43–0.9 mm. The model was initialized with random parameters and it was trained on pairs of downsampled and fully sampled scans obtained from the fastMRI neuro dataset [20, 21]. The dataset consists of 4,469 (2,575 3-T, 1,894 1.5-T) training and 1,378 (775 3-T, 603 1.5-T) validation patients with fully sampled two-dimensional axial brain pre- and post-contrast T1-weighted, T2-weighted FLAIR, and T2-weighted images following the standard fastMRI training/validation split without any exclusion criteria. Pathologies included but were not limited to healthy patients, resection cavities, craniotomy, edema, and nonspecific lesions; however, the exact distribution of the classes on the full fastMRI dataset is not known [23]. The inverse Fourier transform was performed along the *z*-direction prior to DL reconstruction. The dataset has an in-plane resolution between 0.43 mm and 0.9 mm, with the median resolution being 0.6875 × 0.6875 mm^2^.

To emulate clinical iMRI protocol settings, the FOV of the data was first randomly cropped in image space to a quadratic or two-fold oversampled image size along the frequency encoding direction. Subsequently, k-space was randomly cropped to ensure an in-plane image resolution between 1.2 mm and 1.6 mm—matching the native resolution found in the iMRI protocols used in this study. The downsampled k-space was thereafter masked with a Poisson-disc sampling pattern. This pattern was generated randomly by either SigPy [24] or the BART Toolbox [25] with randomized acceleration factors that matched the acceleration factors seen in the clinical protocol, *i.e*., 1.5-, 3.7-, 4-, and 10-times acceleration factors. By utilizing two sampling pattern methods, we aimed to better encompass the proprietary vendor sampling masks. The model was trained to reconstruct two-dimensional axial slices.

The Adam optimizer [26] was used with a cosine annealing learning rate scheduler [27]. The model was trained for a total of 200 epochs with a learning rate ${lr}=1e-3$ to minimize the structural similarity index measure [28], and one epoch iterated over 25,000 training examples. Model evaluation was performed after each epoch, and the epoch with the lowest validation loss, *i.e.*, structural similarity index measure was selected for inference. The training was performed with a batch size of two, utilizing two NVIDIA RTX3090 graphical processing units.

### Reconstruction and image processing

The intraoperative scans were zero-padded in k-space to reach the desired target resolution, reconstructed by the model, and the bias field corrected [29]. Repeated samples were averaged in k-space before DL reconstruction.

In preparation for the quality assessment, the on-scanner images were up-sampled such that their resolution matched the reconstructions. All images were co-registered to the on-scanner T1 pre-contrast image [29] and de-faced before evaluation [30].

**Fig. 1:**
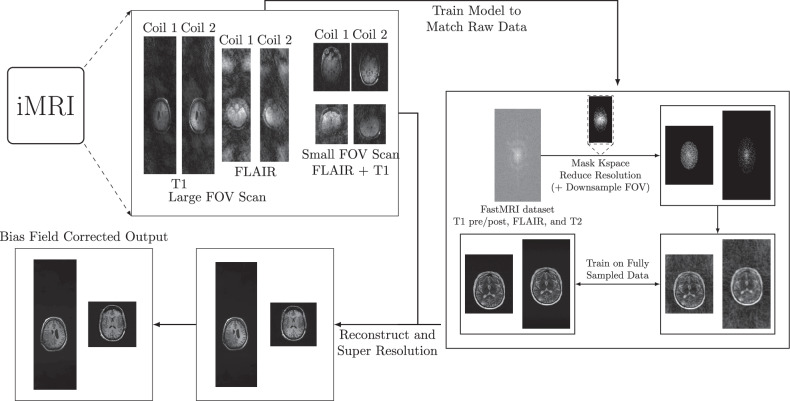
Illustration of the raw iMRI data before any reconstruction, the model training/reconstruction regime, and the resulting DL reconstruction with and without bias field correction. The iMRI scans have two different imaging protocols a large FOV and a small FOV. A DL model was trained on the fastMRI dataset to match the iMRI protocol with respect to the masked k-space, FOVs, and resolution. The model was used to reconstruct the on-scanner iMRI scans followed by bias field correction

An example of the raw protocol images, the training regime, and the reconstruction process is illustrated in Fig. 1.

### Qualitative assessment

Prior to quality assessment, the imaging variants (DL and CS) were randomly assigned with a pseudorandom number generator in Python to “A” or “B” for each patient to ensure blinded assessment. Assessment of “A” and “B” for a given patient was performed at the same time or in a similar timeframe.

Image quality assessment was performed by two neuroradiologists (readers 1 and 2), with 10 years and 19 years of experience, and a neurosurgeon with 8 years of experience (reader 3). The readers were provided with data in Digital Imaging and Communications in Medicine or Neuroimaging Informatics Technology Initiative format, and they had the freedom to choose their preferred reading software. The images were ranked on a Likert scale (1 = nondiagnostic, 2 = poor, 3 = acceptable, 4 = good, and 5 = excellent) based on the following metrics: imaging artifacts, perceived spatial resolution, anatomic conspicuity, diagnostic confidence, SNR, and contrast. In addition, the CS and DL were blindly ranked based on preference using the following scores: 1 = strongly favors A, 2 = favors A, 3 = indifferent, 4 = favors B, and 5 = strongly favors B. A two-sample Wilcoxon signed-rank test was performed to test the statistical significance of the assessment; *p*-values lower than 0.050 were considered significant.

Given the iMRI setup, with the dual surface coil setup, we instructed the readers to solely focus on the peritumoral area, in line with the focus of the intraoperative setting. In addition, readers gave their overall preference based on all the combined imaging data per patient. Note, that the number of MRI sequences per patient varied, so each scoring is based on a global score for the sequences available for that patient.

## Results

The mean and standard deviation for the qualitative assessments of the DL and CS image variants for all sequences available for a given patient from the three expert readers are given in Table 1. There was a significant difference in the scores between DL and CS reconstructed images for all metrics/readers, except for two metrics from reader 3. Across the six evaluation metrics, DL achieved significantly higher scores than CS for both readers 1 and 2, except for “image artifacts” by reader 1. For reader 3, the CS scored significantly better than the DL counterpart for all metrics except contrast and perceived spatial resolution.

**Table 1:** The mean and standard deviation (median) for the qualitative assessment of image artifacts, perceived spatial resolution, anatomic conspicuity, diagnostic confidence, SNR, and contrast from three expert readers on DL and CS reconstructed images

Feature	Reader 1			Reader 2			Reader 3		
	DL	CS	*p*-value	DL	CS	*p*-value	DL	CS	*p*-value
Image artifacts	4.5 ± 0.7 (5)	4.7 ± 0.7 (5)	0.0330	4.5 ± 0.8 (5)	3.9 ± 0.6 (4)	0.009	3.3 ± 0.9 (3)	3.9 ± 1.0 (4)	0.001
Perceived spatial resolution	4.9 ± 0.4 (5)	3.4 ± 0.5 (3)	< 0.001	4.95 ± 0.2 (5)	3.9 ± 0.5 (4)	< 0.001	3.8 ± 1.0 (4)	3.8 ± 0.7 (4)	0.752
Anatomic conspicuity	4.9 ± 0.3 (5)	3.8 ± 0.5 (4)	< 0.001	4.95 ± 0.2 (5)	4.0 ± 0.3 (4)	< 0.001	3.5 ± 0.8 (4)	3.7 ± 0.6 (4)	0.021
Diagnostic confidence	4.8 ± 0.4 (5)	4.0 ± 0.3 (4)	< 0.001	4.9 ± 0.4 (5)	3.9 ± 0.5 (4)	< 0.001	3.5 ± 0.8 (4)	3.7 ± 0.6 (4)	0.021
SNR	4.6 ± 0.7 (5)	3.6 ± 0.6 (4)	< 0.001	4.9 ± 0.4 (5)	4.5 ± 0.6 (5)	0.003	3.3 ± 0.8 (4)	3.7 ± 0.7 (4)	0.006
Contrast	4.8 ± 0.6 (5)	3.7 ± 0.6 (4)	< 0.001	4.95 ± 0.3 (5)	4.5 ± 0.6 (5)	< 0.001	3.7 ± 0.8 (4)	3.9 ± 0.5 (4)	0.167

Due to the coil setup, the area of assessment was the area of resection and peritumoral

**Fig. 2:**
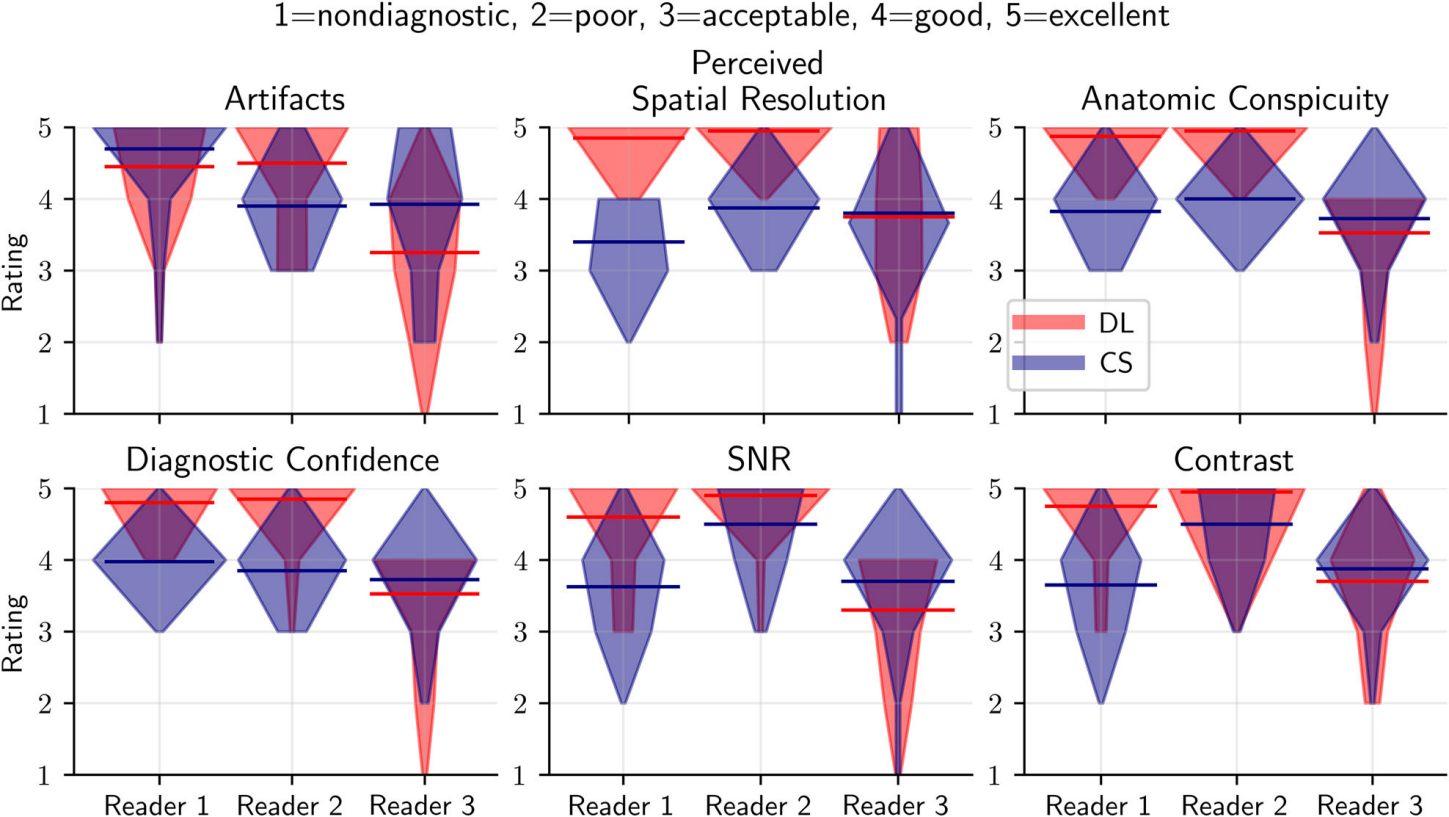
A violin plot of the qualitative assessment of the DL and CS reconstructions for 40 intraoperative patients from three expert readers. The mean of the DL and CS assessments are shown with the colored lines. No smoothing has been used in the violin plot due to the discrete nature of the assessment

Averaged across all metrics, the DL reconstructions were rated with a higher score than CS reconstruction for 72%, 72%, and 14% of the ratings, for readers 1, 2, and 3, respectively. Conversely, the CS reconstruction was preferred over DL reconstruction for 8%, 7%, and 37% of the ratings, respectively for readers 1, 2, and 3. The violin plot distribution of the qualitative assessments is shown in Fig. 2. Of note, since the assessment criteria were discrete, no interpolation between the metrics was performed.

**Fig. 3:**
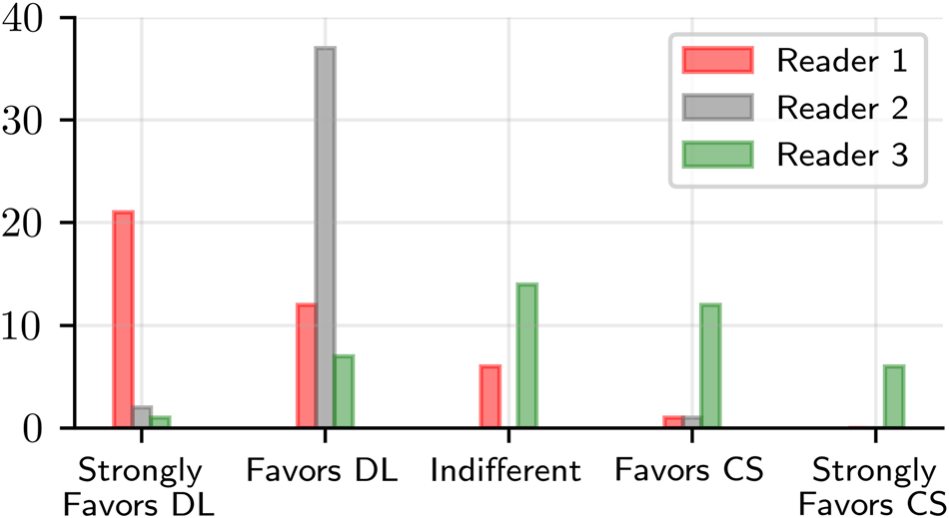
The preferred reconstruction variant among the three expert readers. DL, Deep learning; CS, Compressed sense

The distribution of the preferred reconstruction is given in Fig. 3. The DL reconstruction was favored or strongly favored in 33/40, 39/40, and 8/40 of the cases for readers 1, 2, and 3, respectively. The CS reconstructions were favored or strongly favored in 1/40, 1/40, and 18/40 of the cases for readers 1, 2, and 3, respectively.

**Fig. 4:**
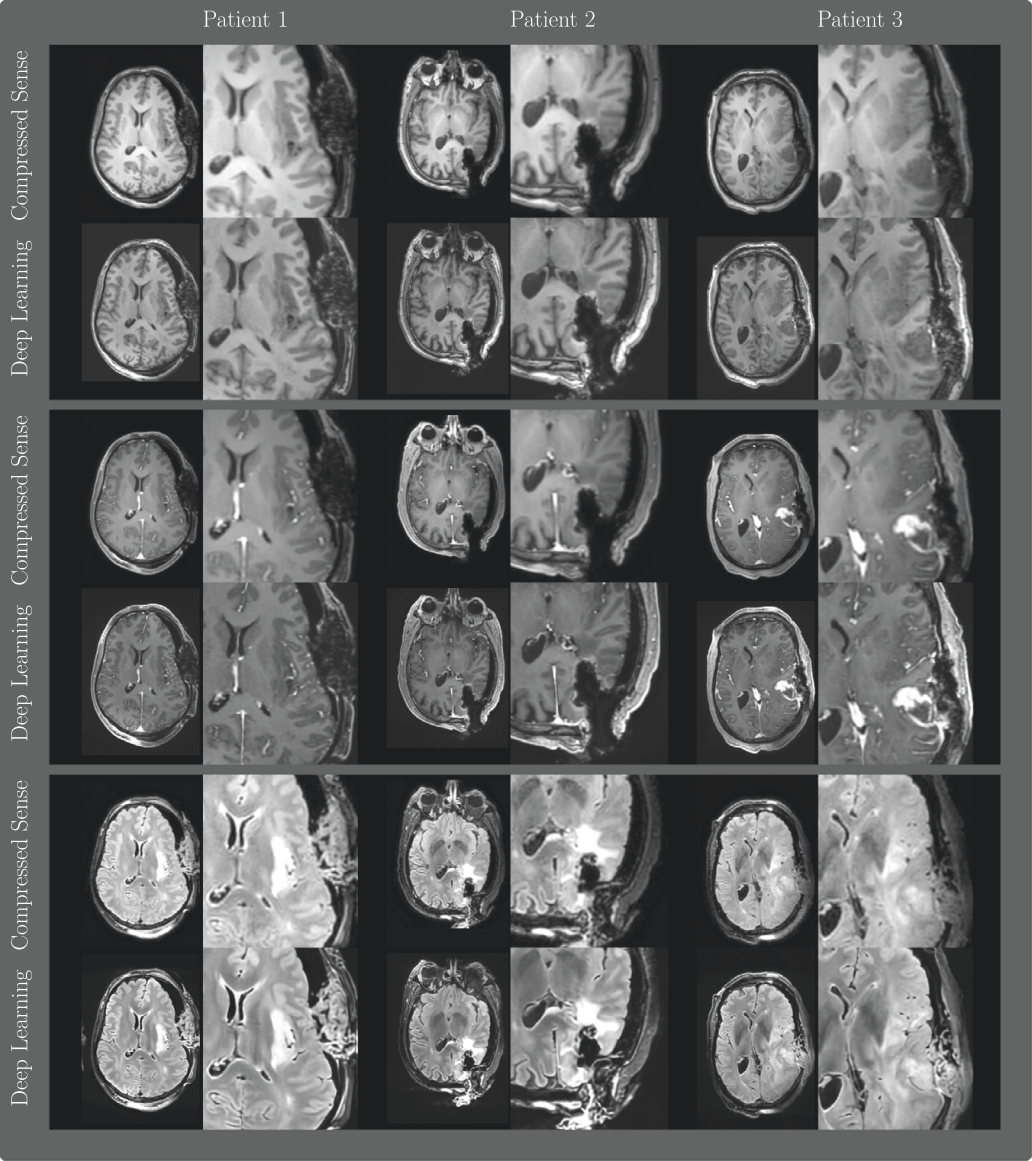
Representative examples of the DL and CS reconstructions from four different patients, depicting T1-weighted pre-contrast, T1-weighted post-contrast, and T2-weighted FLAIR scans. Window level was chosen between 0.05 and 0.995 percentiles

**Fig. 5:**
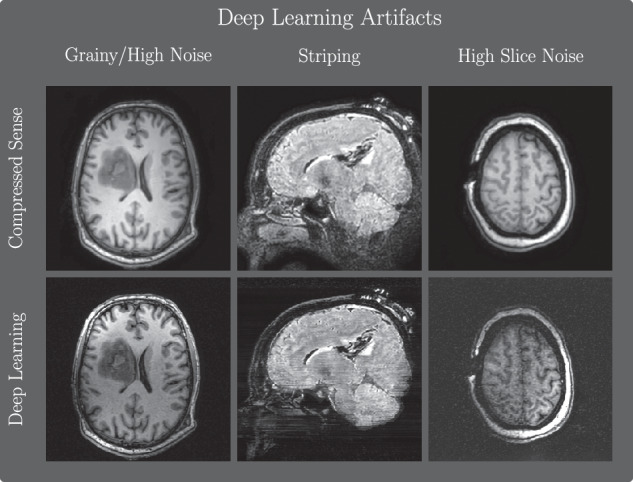
Examples of DL-specific artifacts. This includes high noise levels for a given scan, striping-like artifacts, and high noise for one slice in an image

Representative images of the DL and CS reconstructions are displayed in Fig. 4. We note that the DL reconstructions show a higher level of detail in the resection area, most pronounced in the T2-weighted FLAIR images. In some cases, imaging artifacts were reported by the readers, and DL-specific artifacts are shown in Fig. 5. In particular, the most common artifacts mentioned were high noise/grainy images and reduced signal distance from the receiver coils. Upon later inspection by the authors, we noted that the high noise artifact can be classified into two categories: whole brain noise and slice-specific high noise levels.

## Discussion

In this study, we have adapted and trained a DL reconstruction network to reconstruct on-scanner undersampled iMRI data. The model was qualitatively evaluated and compared to the CS reconstructions by three expert readers on a cohort of 40 patients who underwent iMRI. DL was found to reconstruct iMRI with significantly better image quality compared to CS for two of the three readers. Notably, the neuroradiologists (readers 1 and 2) favored overall DL over CS to a larger extent than the neurosurgeon (reader 3). Reader 3 assessed that the CS reconstruction had improved image quality compared to the DL equivalent. The difference in image quality assessment reflects the subjective nature of the interpretation. Further, iMRI is different from conventional MRI diagnostics in that radiologists rarely review these images whereas they are a central part of the neurosurgeon’s intraoperative assessment of the extent of tumor resection. In discussing the results with the expert reviewers, it was noted that the focus of the neurosurgeon was mainly on the extent of resection, whereas the radiologist focused more on image quality and diagnostic quality in the area of interest.

iMRI requires specialized operating and scanning equipment [8], relying on the use of flexible surface coils to adjust to the required head position dictated by the tumor location. The required coil setup typically results in low SNR in areas distant from the target tumor region. This is partially compensated for by the coil sensitivity maps used in the CS reconstruction, but not the DL variants. As such, the low SNR artifact was particularly visible in the DL images, as pointed out by reader 3. The low intensity coupled with the two-dimensional nature of the reconstruction gave rise to pronounced striping artifacts in some cases with DL reconstruction. It is worth noting that most of these artifacts occurred outside the resection area of interest due to the coil placement. The use of three-dimensional reconstruction could potentially reduce or completely remove the striping artifact and the rarely seen slice-wise noise since the network would not be dependent on slice-wise variability. Despite the artifacts, the DL model showcased high-quality reconstruction and super-resolution, and all reviewers commented that the DL reconstructed images were in general of good or acceptable quality. Further improvements should focus on reducing the striping artifact and improving the signal intensity in the low-intensity regions caused by the iMRI imaging setting.

**Fig. 6:**
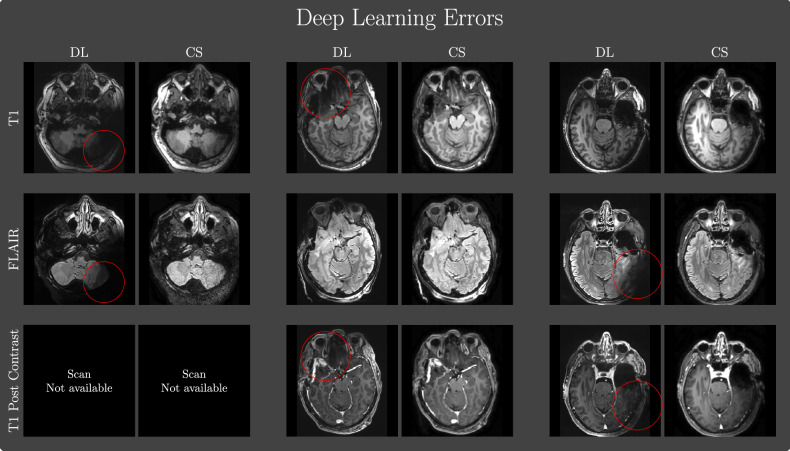
Three cases highlighted by the neurosurgeon where the DL reconstructions had considerable artifacts and were evaluated as nondiagnostic or poor by the neurosurgeon compared to the compressed sensing (CS) counterpart. For the two last patients, the artifacts affected the area of resection for one or more of the scans. The first patient, *i.e.*, columns 1 and 2 did not have a T1-weighed post-contrast series

Importantly, DL-specific artifacts were rare, but in some cases worrisome when brain regions close to the area of resection had low signal as can be seen in Fig. 6 which highlights three cases with worrisome DL-related artifacts. Note, the artifact in the first case stems from the bias-field correction. The two remaining cases had reduced signal in the area of resection in one or more of the scans taken. Upon close inspection of the raw non-reconstructed images, it was seen that the affected scans either had a high degree of noise or a very low signal in the affected area. Although the artifacts were a rare occurrence, it is important to be addressed as they can impact surgical decision-making.

The majority of works in DL-based reconstruction of MRI have focused on retrospectively downsampled data. Recent studies have demonstrated that DL can successfully be adapted to on-scanner downsampled data, particularly in contexts with relatively normal pathology and scanner configurations [15–18]. This study aims to validate the generalizability of DL reconstruction of highly non-conventional brain images.

Except for imaging artifacts, DL reconstruction significantly outperformed the CS reconstruction on all assessment metrics by both readers 1 and 2. In addition, since the fastMRI dataset was imaged exclusively on Siemens scanners [20, 21], this study implies that DL models exhibit generalizability across vendors and scanner setups even on an unordinary scanner setup that is iMRI. Nonetheless, it is reasonable to expect that further improvements could be made if the training dataset better represented the characteristics in the iMRI data with similar coil elements, data from the same vendor, and similar FOV. We opted to use both the 1.5-T and 3-T fastMRI data to better encompass different image characteristics to allow for improved generalizability although the iMRI data was acquired on a 3-T scanner.

A concern of iMRI is the increased procedure time and risk of infection [31]. DL-based reconstruction methods have shown promise to decrease the total scan time by 45% and 60% [15, 18] over traditional acceleration methods on standardized imaging setups. Nonetheless, due to the challenging iMRI imaging setup, this study aimed to determine whether DL reconstruction could improve image quality and successfully adapt to the iMRI imaging setup and showed promising results by two of the three readers. Improved image quality could further help the neurosurgeon to distinguish between residual tumors, artifacts, and contrast leakage from the surgical cavity. Incidentally, this was the major concern raised by reader 3. Based on the results of this study, we believe DL-based reconstruction in iMRI could lead to easier-to-interpret images, however, further studies are needed to validate this since the number of studies that focus on DL reconstruction for iMRI is limited.

The findings in this study should be interpreted with multiple limitations in mind. First, the study included a relatively limited patient cohort size of 40 patients from a single site. Additional patients from multiple sites would need to further validate the generalizability of the model. Second, the DL reconstructions were only compared to their respective CS reconstruction, and not the ground truth in a quantitative analysis. Acquiring fully sampled ground truth images in an iMRI setting would be very time-consuming and therefore not feasible for ethical reasons and the clinically used CS-based accelerated reconstruction was therefore considered the best standard of comparison. Third, the number of MRI sequences per patient varied, so each scoring is not for the same number of sequences. We opted for this method of assessment since an expert reader would evaluate the case “as a whole” with all scans. Fourth, although the readers were blinded, and the DL/CS scans were randomized per patient; it is feasible to accurately guess which is which by an expert reader due to the general imaging characteristics of the different reconstruction methods. Lastly, additional neurosurgeon readers would further strengthen the findings of this study, potentially affect the conclusion, and help quantify the difference in opinion between the readers.

Future work could explore the effect of a further increase in acceleration factor. Here, we opted against retrospectively downsampling the on-scanner downsample data to simulate further acceleration as we wanted the study to solely focus on on-scanner undersampled data. To properly evaluate the potential gains from the improved image quality, a “during surgery” evaluation would strengthen the findings in this study. Additional focus should be placed on the evaluation of the extent of resection and quantizing differences in tumor residuals.

In conclusion, DL-based iMRI reconstruction allows for high-quality reconstructions of on-scanner undersampled iMRI. The neuroradiologists favored the DL reconstruction over the CS counterpart, while the neurosurgeon favored the CS reconstruction. Further work is needed to account for low coil sensitivity distant from the area of resection and alleviate the striping artifact seen from the two-dimensional nature of the reconstruction.

## Supplementary information

ELECTRONIC SUPPLEMENTARY MATERIAL

## Data Availability

The datasets used for this study are available from the corresponding author upon reasonable request. This work was available as a pre-print at 10.48550/arXiv.2401.12771.

## Abbreviations

CS: Compressed sense
DL: Deep learning
FLAIR: Fluid-attenuated inversion-recovery
FOV: Field-of-view
iMRI: Intraoperative magnetic resonance imaging
SNR: Signal-to-noise ratio

## Footnotes

1 The model weights and training scripts are available at https://github.com/JonOttesen/intraop_recon
