# Supplementary material for: Deep learning-based Intraoperative MRI reconstruction
Source: Eur Radiol Exp. 2025 Feb 25;9:29. doi: 10.1186/s41747-024-00548-9 (PMC11861787; doi:10.1186/s41747-024-00548-9)
Supplement: Supplementary file 1 — ELECTRONIC SUPPLEMENTARY MATERIAL [file 41747_2024_548_MOESM1_ESM.pdf]

Deep Learning-based Intraoperative MRI Reconstruction

ELECTRONIC SUPPLEMENTARY MATERIAL

Eur Radiol Exp (2025) Ottesen JA, Storas T, Vatnehol SAS, et al.

Subject	A or B	Imaging Artifacts	Perceived Spatial Resolution (Sharpness) (1-5)	Anatomic Conspicuity	Diagnostic Confidence	Signal To Noise	Contrast	Preference [A/B] (Relative scoring: 1 strongly favours series A, 2 favours series A, 3 indifferent, 4 favours series B, 5 strongly favours series B)
		1 = nondiagnostic, 2 = poor, 3 = acceptable, 4 = good, 5 = excellent						
		General	Area Of Resection / Peritumoral region					
Reader 1								
Patient 1	CS	5	4	4	4	4	4	5
	DL	5	5	5	5	5	5	
Patient 2	DL	5	5	5	5	5	5	1
	CS	5	3	3	4	3	4	
Patient 3	CS	5	3	3	4	3	4	5
	DL	5	5	5	5	5	5	
Patient 4	CS	5	3	4	4	3	4	5
	DL	5	5	5	5	5	5	
Patient 5	CS	5	3	4	4	3	3	5
	DL	5	5	5	5	5	5	
Patient 6	DL	5	5	5	5	5	5	1
	CS	5	3	3	4	3	3	
Patient 7	CS	5	3	3	4	4	4	5
	DL	5	5	5	5	5	5	
Patient 8	CS	4	4	4	4	4	4	5
	DL	4	5	5	5	5	5	
Patient 9	CS	5	3	4	4	3	3	5
	DL	4	5	5	5	5	5	
Patient 10	DL	5	5	5	5	5	5	1

	CS	5	3	4	4	3	3	
Patient 11	CS	5	3	4	4	3	3	5
	DL	4	5	5	5	5	5	
Patient 12	CS	5	4	4	4	4	4	4
	DL	4	5	5	5	5	5	
Patient 13	CS	5	3	3	4	3	3	5
	DL	5	5	5	5	5	5	
Patient 14	CS	5	3	3	4	3	3	5
	DL	5	5	5	5	5	5	
Patient 15	DL	3	5	5	5	5	5	1
	CS	4	3	3	4	3	3	
Patient 16	CS	5	3	4	4	3	3	5
	DL	4	5	5	5	5	5	
Patient 17	DL	4	4	4	4	3	3	2
	CS	3	3	3	3	3	3	
Patient 18	DL	4	5	5	5	5	5	2
	CS	5	4	4	4	4	4	
Patient 19	DL	2	4	4	4	4	3	3
	CS	3	4	4	4	4	3	
Patient 20	CS	5	3	4	4	4	4	4
	DL	5	5	5	5	4	5	
Patient 21	DL	5	5	5	5	5	5	1

	CS	5	4	4	4	4	4	
Patient 22	CS	5	3	4	4	3	3	4
	DL	4	5	5	5	4	5	
Patient 23	DL	4	4	4	4	3	3	1
	CS	2	2	3	3	2	2	
Patient 24	DL	4	5	5	5	5	5	1
	CS	5	4	4	4	4	4	
Patient 25	DL	5	5	5	5	5	5	1
	CS	4	4	4	4	4	4	
Patient 26	DL	5	5	5	5	5	5	1
	CS	5	3	4	4	4	3	
Patient 27	DL	4	5	5	5	4	5	3
	CS	5	4	5	5	4	5	
Patient 28	DL	5	5	5	5	4	5	3
	CS	5	4	5	4	5	5	
Patient 29	DL	5	5	5	5	5	5	1
	CS	5	3	4	4	4	4	
Patient 30	DL	5	5	5	5	5	5	2
	CS	5	4	4	4	5	4	
Patient 31	CS	5	4	4	4	4	4	4
	DL	4	5	5	5	5	5	
Patient 32	CS	5	3	4	4	4	4	4

	DL	5	5	5	5	5	5	
Patient 33	DL	5	4	5	4	3	4	3
	CS	5	4	4	4	4	4	
Patient 34	DL	4	5	5	5	5	5	2
	CS	5	3	4	4	3	4	
Patient 35	DL	3	5	5	4	5	5	2
	CS	4	4	4	4	4	4	
Patient 36	CS	5	4	4	4	4	4	4
	DL	4	5	5	5	5	5	
Patient 37	DL	4	4	4	4	3	4	3
	CS	5	3	4	4	4	4	
Patient 38	CS	5	4	4	4	4	4	4
	DL	5	5	5	5	5	5	
Patient 39	CS	4	3	4	4	4	3	3
	DL	5	4	5	4	3	4	
Patient 40	CS	5	4	4	4	4	4	2
	DL	5	5	4	4	4	4	

Reader 2

Patient 1	CS	4	4	4	4	5	5	4
	DL	4	5	5	5	5	5	
Patient 2	DL	4	5	5	5	5	5	2

	CS	4	4	4	4	5	5	
Patient 3	CS	4	4	4	4	5	5	4
	DL	5	5	5	5	5	5	
Patient 4	CS	3	3	3	3	3	3	5
	DL	5	5	5	5	5	5	
Patient 5	CS	3	3	3	3	3	3	5
	DL	5	5	5	5	5	5	
Patient 6	DL	4	5	5	5	5	5	2
	CS	3	3	4	4	3	5	
Patient 7	CS	4	4	4	3	5	5	4
	DL	5	5	5	4	5	5	
Patient 8	CS	4	4	4	4	5	5	4
	DL	5	5	5	5	5	5	
Patient 9	CS	3	4	4	4	5	5	4
	DL	5	5	5	5	5	5	
Patient 10	DL	4	5	5	5	5	5	2
	CS	4	4	4	4	5	5	
Patient 11	CS	4	4	4	4	5	5	4
	DL	5	5	5	5	5	5	
Patient 12	CS	4	4	4	4	5	5	4
	DL	5	5	5	5	5	5	
Patient 13	CS	4	4	4	5	5	5	4

	DL	5	5	5	5	5	5	
Patient 14	CS	4	3	4	3	5	5	4
	DL	5	5	5	5	5	5	
Patient 15	DL	5	5	5	5	5	5	2
	CS	3	4	4	4	4	4	
Patient 16	CS	4	3	4	3	5	5	4
	DL	5	5	5	5	5	5	
Patient 17	DL	4	5	5	5	5	5	2
	CS	3	4	4	3	4	4	
Patient 18	DL	5	5	5	5	5	5	2
	CS	4	4	4	4	4	4	
Patient 19	DL	5	5	5	5	5	5	2
	CS	3	4	4	3	4	4	
Patient 20	CS	4	4	4	4	5	5	4
	DL	5	5	5	5	5	5	
Patient 21	DL	5	5	5	5	5	5	2
	CS	4	4	4	4	4	4	
Patient 22	CS	4	4	4	4	5	5	4
	DL	5	5	5	5	5	5	
Patient 23	DL	5	5	5	4	4	4	2
	CS	3	4	4	3	3	3	
Patient 24	DL	5	5	5	5	5	5	2

	CS	3	4	4	3	4	4	
Patient 25	DL	5	5	5	5	5	5	2
	CS	4	4	4	4	4	4	
Patient 26	DL	5	5	5	5	5	5	2
	CS	4	4	4	4	4	4	
Patient 27	DL	3	5	5	4	4	5	2
	CS	3	3	4	4	5	4	
Patient 28	DL	3	5	5	4	4	5	2
	CS	3	3	4	4	5	4	
Patient 29	DL	5	5	5	5	5	5	2
	CS	4	4	4	4	4	4	
Patient 30	DL	5	5	5	5	5	5	2
	CS	4	4	4	4	4	4	
Patient 31	CS	4	4	4	4	5	5	4
	DL	5	5	5	5	5	5	
Patient 32	CS	4	4	4	4	5	5	4
	DL	5	5	5	5	5	5	
Patient 33	DL	4	4	4	4	5	5	4
	CS	5	5	5	5	5	5	
Patient 34	DL	5	5	5	5	5	5	2
	CS	4	4	4	4	4	4	
Patient 35	DL	3	5	5	5	5	5	2

	**CS**	**5**	**4**	**4**	**4**	**4**	**4**	
**Patient 36**	**CS**	**4**	**4**	**4**	**4**	**5**	**5**	**4**
	**DL**	**5**	**5**	**5**	**5**	**5**	**5**	
**Patient 37**	**DL**	**3**	**5**	**5**	**5**	**5**	**5**	**2**
	**CS**	**5**	**4**	**4**	**4**	**4**	**4**	
**Patient 38**	**CS**	**5**	**4**	**4**	**4**	**5**	**5**	**4**
	**DL**	**3**	**5**	**5**	**5**	**5**	**5**	
**Patient 39**	**CS**	**5**	**4**	**4**	**4**	**5**	**5**	**4**
	**DL**	**3**	**5**	**5**	**5**	**5**	**5**	
**Patient 40**	**CS**	**4**	**4**	**4**	**4**	**5**	**5**	**4**
	**DL**	**5**	**5**	**5**	**5**	**5**	**5**	

Reader 3

**Patient 1**	**CS**	**4**	**3**	**3**	**4**	**3**	**4**	
	**DL**	**3**	**4**	**4**	**4**	**3**	**4**	**2**
**Patient 2**	**DL**	**2**	**5**	**4**	**4**	**4**	**4**	
	**CS**	**4**	**3**	**4**	**4**	**3**	**3**	**2**
**Patient 3**	**CS**	**3**	**3**	**4**	**4**	**3**	**3**	
	**DL**	**3**	**4**	**4**	**4**	**4**	**4**	**4**
**Patient 4**	**CS**	**3**	**3**	**4**	**4**	**3**	**3**	
	**DL**	**2**	**4**	**4**	**4**	**4**	**4**	**2**
**Patient 5**	**CS**	**3**	**3**	**4**	**4**	**4**	**4**	**3**

	DL	4	4	4	4	3	4	
Patient 6	DL	4	4	4	4	4	4	2
	CS	4	3	4	4	3	3	
Patient 7	CS	4	3	4	3	4	3	4
	DL	4	5	4	4	4	4	
Patient 8	CS	5	4	5	5	4	5	1
	DL	4	4	4	4	4	3	
Patient 9	CS	4	4	4	4	4	4	3
	DL	3	5	4	4	4	3	
Patient 10	DL	2	4	4	4	5	4	4
	CS	5	3	4	4	4	5	
Patient 11	CS	4	5	4	4	4	4	4
	DL	4	5	4	4	4	5	
Patient 12	CS	5	4	4	4	4	4	3
	DL	2	5	4	4	4	4	
Patient 13	CS	5	4	4	4	4	4	3
	DL	4	5	4	4	4	4	
Patient 14	CS	5	4	4	4	4	4	3
	DL	4	5	4	4	4	4	
Patient 15	DL	2	4	3	3	4	4	4
	CS	4	4	3	3	4	5	
Patient 16	CS	5	4	4	4	4	4	3

	DL	3	5	4	4	4	5	
Patient 17	DL	2	2	2	2	2	2	3
	CS	2	1	2	2	2	2	
Patient 18	DL	3	4	4	4	4	4	3
	CS	4	4	4	4	4	4	
Patient 19	DL	1	4	2	2	2	2	5
	CS	2	4	3	3	4	4	
Patient 20	CS	4	4	4	4	4	4	2
	DL	3	3	4	4	3	4	
Patient 21	DL	4	3	4	4	4	4	3
	CS	5	4	4	4	4	4	
Patient 22	CS	4	4	4	4	4	4	5
	DL	2	3	4	4	2	2	
Patient 23	DL	2	3	2	2	2	2	3
	CS	3	4	2	2	1	3	
Patient 24	DL	3	4	4	4	3	4	4
	CS	4	4	4	4	4	4	
Patient 25	DL	4	5	4	4	4	4	3
	CS	4	4	4	4	4	4	
Patient 26	DL	4	3	4	4	3	4	4
	CS	5	4	4	4	4	4	
Patient 27	DL	3	3	4	4	3	3	3

	CS	4	4	4	4	4	4	
Patient 28	DL	3	4	4	4	4	4	4
	CS	4	5	4	4	4	4	
Patient 29	DL	4	5	4	4	4	4	3
	CS	4	4	4	4	4	4	
Patient 30	DL	4	3	4	4	4	4	4
	CS	5	4	4	4	4	5	
Patient 31	CS	5	4	4	4	4	4	3
	DL	4	4	4	4	4	4	
Patient 32	CS	5	4	3	3	3	4	4
	DL	4	5	3	3	4	4	
Patient 33	DL	4	5	4	4	4	4	2
	CS	5	4	4	4	3	4	
Patient 34	DL	2	2	2	2	2	4	5
	CS	5	3	3	3	3	4	
Patient 35	DL	3	2	3	3	3	3	5
	CS	4	4	4	4	4	4	
Patient 36	CS	4	3	3	3	3	3	1
	DL	2	2	1	1	1	3	
Patient 37	DL	3	3	3	3	2	4	5
	CS	4	4	4	4	4	4	
Patient 38	CS	4	3	3	3	3	4	2

	DL	3	4	2	2	2	4	
Patient 39	CS	3	4	3	3	4	4	2
	DL	3	3	3	3	3	3	
Patient 40	CS	4	4	4	4	4	4	2
	DL	3	3	3	3	3	4	
